# Alterations of the Hippocampal Networks in Valproic Acid-Induced Rat Autism Model

**DOI:** 10.3389/fncir.2022.772792

**Published:** 2022-02-04

**Authors:** Veronika Bódi, Tímea Májer, Viktor Kelemen, Ildikó Világi, Attila Szűcs, Petra Varró

**Affiliations:** Ex vivo Electrophysiology Laboratory, Department of Physiology and Neurobiology, Institute of Biology, Eötvös Loránd University, Budapest, Hungary

**Keywords:** VPA, autism (ASD), electrophysiology, patch clamp, hippocampus

## Abstract

Autism Spectrum Disorder (ASD) is one of the most frequently diagnosed neurodevelopmental disorders, characterized among others by impairments in social interactions and repetitive behavior. According to one of the leading hypotheses about its origin, ASD is caused by the imbalance of excitatory and inhibitory circuit activity. ASD-related morphological and functional changes can be observed in several brain regions i.e., in the prefrontal cortex and the hippocampus. It is well-established that prenatal valproic-acid (VPA) exposure of rats on day 12.5 leads to neurodevelopmental alterations with autism-like clinical and behavioral symptoms. The aim of this study was to investigate potential changes in the excitability of neuronal networks and individual neurons of the hippocampus elicited by prenatal VPA treatment. As there are marked sex differences in ASD, offspring of both sexes were systematically tested, using two different age groups, to elucidate eventual differences in neurodevelopment after VPA treatment. Excitatory connections and long-term synaptic plasticity as well as intrinsic excitability of CA1 pyramidal cells were examined. Pregnant female Wistar rats received saline or 500 mg/kg VPA i. p. on gestation day 12.5. Brain slices of 6-week-old and 3-month-old offspring were investigated using extra- and intracellular electrophysiological techniques. Field potential- and whole-cell patch clamp recordings were carried out to measure network excitability and single cell activity in the CA1 region hippocampus. Enhanced excitability of hippocampal networks was detected in the 6-week-old VPA-treated male rats; however, this change could not be observed in 3-month-old males. Intrinsic excitability of single neurons, however, was increased in 3-month-old males. In 6-week-old treated females, the most prominent effect of VPA was an increase in voltage sag, to a similar degree to the neurons of the older age group. In 3-month-old females, a network excitability increase could be demonstrated, in a lesser degree than in younger males. It can be concluded, that VPA treatment had diverse effects on hippocampal excitability depending on the sex and the age of the animals. We found that certain alterations manifested in 6-week-old rats were compensated later, on the other hand, other changes persisted until the age of 3 months.

## Introduction

Autism Spectrum Disorder (ASD) is frequently diagnosed as a neurodevelopmental disorder with a prevalence of 1-1.7% (Young et al., 2018; Mohammadi et al., 2020). ASD is characterized by social communication and interaction impairments, restricted interests and repetitive behaviors, however, the manifestation of the symptoms is quite heterogeneous (Scheggi et al., 2020). Currently, the pathogenesis is not properly understood, but it is generally assumed that both genetic and environmental factors play a pivotal role in disordered pre- and postnatal brain development (Nicolini and Fahnestock, 2018; Li et al., 2019). Genetic animal models of ASD usually display an excitatory/inhibitory (E/I) imbalance caused by the abnormal development of excitatory and inhibitory connections in the brain (Rhee et al., 2018).

Many brain regions in autism exhibit abnormalities including decreased Purkinje cell counts and atrophy in the cerebellum, changes in temporal lobe activity and delayed sensorimotor development (Hou et al., 2018). According to the manifestation of symptoms, it is assumed that the medial prefrontal cortex and the hippocampus (HC) are among the most affected brain areas related to ASD. The HC plays roles in several cognitive processes including memory, spatial navigation and the emotional experience of stress. Furthermore, the HC and its associated cortices are involved in future thinking, decision making, problem solving and choice-guided behavior. In children, higher volumes of HC have been associated with more developed cognitive abilities, expressive language and memory skills (Reinhardt et al., 2020). According to MRI studies, the development pattern of HC in ASD is different from typically developing individuals. Increase or reduction was found in the hippocampal volumes of patients with ASD according to the age and sex of the individuals (Li et al., 2019; Xu et al., 2020), moreover, different subtypes were found among children with ASD related to the overall brain size (Reinhardt et al., 2020). Examination of abnormalities in human brains has its limits; MRI, as a non-invasive method is a key technology in autism research (Li et al., 2019), however, to investigate the background mechanisms of ASD in details, animal models should be involved. In rodent models of ASD, the loss of pyramidal cells in the CA1 and CA3 region of the HC was reported. GABAergic system dysregulation, including decreased number of GABAergic interneurons and impaired GABAergic neurotransmission has been proposed as a source of E/I imbalance in the HC, as well (Hou et al., 2018).

The valproic acid (VPA) model is a well-established animal model of ASD that can display the core symptoms of the disorder (Hou et al., 2018; Ruhela et al., 2019). VPA is a drug commonly prescribed for patients with epilepsy, however, it is also a potent teratogen increasing the risk of giving birth to an autistic child (Hou et al., 2018). In rats, prenatal VPA exposure on embryonic day 12.5 is also associated with high risk of ASD in the offspring, as it leads to neurodevelopmental aberration with autism-like symptoms (Zhang et al., 2018; Ruhela et al., 2019).

Since the symptoms of ASD are very heterogeneous and there are no adequate clinical test or treatment available for all patients, the research on the cellular and network level mechanisms in the brain is most warranted. As a further complication, there is a marked sex difference in ASD occurrence with an estimated male:female ratio of 4:1 (Nicolini and Fahnestock, 2018; Young et al., 2018). Accordingly, in rats, severe autism-like behavior deficits were only observed in male offspring after VPA exposure (Young et al., 2018). The aim of this study, therefore, is to investigate the mechanisms of altered excitability linked to ASD with a detailed analysis of the electrophysiological activity of individual pyramidal cells and of networks in the HC in both sexes, in two age groups to elucidate eventual sex differences in the neurodevelopmental effects of VPA. The efficacy and plasticity of excitatory synaptic connections, as well as intrinsic excitability of CA1 pyramidal neurons is studied.

## Materials and Methods

### Animal Maintenance and Treatment

Female Wistar rats (Toxi-coop Ltd., Budapest, Hungary) and their offspring (*n* = 87) were used for the experiments. Experiments were carried out in accordance with the Hungarian Act of Animal Care and Experimentation (1998, XXVIII) and with the **directive 2010/63/EU of the European Parliament and of the Council of 22 September 2010 on the protection of animals used for scientific purposes.** Experimental protocols were approved by the Animal Care and Use Committee of Eötvös Loránd University and Budapest Animal Health Care Authority (license no. PE/EA/772-7/2020). All possible efforts were made to minimize the number of animals used and to minimize animal suffering. Rats were kept under constant 12 h light/dark cycle and controlled temperature (22 ± 2°C). Standard pellet food and tap water were available *ad libitum*.

### Chemicals

Dams of the ASD group offspring received a single intraperitoneal injection of sodium VPA dissolved in 0.9% saline at a dose of 500 mg/kg bw (concentration of the solution was 150 mg/ml) on gestation day 12.5. Dams of the control group received saline at the same time of gestation (Rinaldi, 2008; Nicolini and Fahnestock, 2018; Ruhela et al., 2019). All the compounds were purchased from Sigma-Aldrich Ltd. (Budapest, Hungary).

### Preparation of Brain Slices

Six-week-old and 3-month-old male and female offspring were decapitated under deep chloral-hydrate anesthesia, the brains were isolated and cut with a vibratome (Electron Microscopy Sciences, Hatfield, United States). 400 μm thick hippocampal slices were incubated for at least an hour at room temperature in artificial cerebrospinal fluid (ACSF) oxygenated with carbogen. The composition of ACSF was (in mM): 126 NaCl; 26 NaHCO_3_; 1.8 KCl; 1.25 KH_2_PO_4_; 1.3 MgSO_4_; 2.4 CaCl_2_; 10 glucose, pH 7.2-7.4. For recording, slices were placed in recording chambers held at 33 ± 1°C and through which ACSF bubbled with carbogen was perfused. In case of patch clamp experiments, a submerged-type chamber, while for field potential measurements, a Haas-type interface chamber was used.

### Whole-Cell Patch Clamp Recordings

**FIGURE 1:**
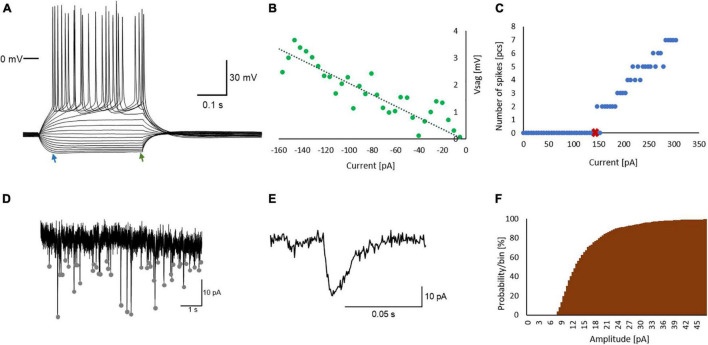
Experimental protocols used in whole-cell patch clamp measurements in CA1 pyramidal neurons. **(A)** Representative voltage responses to gradually increasing current steps. Voltage sag (Vsag) may appear at negative current steps and is described as the difference between the most negative point (blue arrow) and the end (green arrow) of the voltage response to the given step **(B)** Vsag plotted as a function of the injected current. With linear regression a line can be drawn (dotted line) through the data points. The slope of the line is used here as the Vsag index. **(C)** I-V curve; the number of spikes is plotted as a function of the injected current. The current evoking the first spike is the rheobase (red x). **(D)** Representative spontaneous excitatory postsynaptic currents, EPSCs (the autodetected events are marked with gray dots), **(E)** an EPSC and **(F)** a cumulative histogram of EPSC amplitudes.

The experiments were performed in whole-cell patch clamp conditions using a MultiClamp 700B amplifier (Molecular Devices). Signal were filtered (low-pass 6 kHz for current step recordings and low-pass 2.5 kHz for EPSC recording) and recorded (sampling rate: 20 kHz) with a custom-written software in Dasylab. The patch electrodes were pulled from borosilicate glass, had 6-8 MΩ ohmic resistance and were filled with the following solution (in mM): K-gluconate 100, KCl 10, KOH 10, MgCl_2_ 2, NaCl 2, HEPES 10, EGTA 0.2, D-glucose 5; the pH was set to 7.3. Current step protocols consisted of 350-ms-long constant level current steps from −160 pA to 300 pA and incremented by 5 pA until robust firing responses were reached in the CA1 pyramidal cells. The current threshold of spiking was used to determine rheobase. The input-output curve of the neuron was obtained, and spikes counted for each current level (Figures 1A,C). Physiological parameters extracted from the sub- and suprathreshold voltage traces included the resting membrane potential, membrane resistance, spike threshold, rheobase, spike amplitude and cumulative spike number (total number of spikes measured in positive steps). Voltage sag index was also calculated, as the slope of the linear fit of the voltage sag value plotted against the hyperpolarizing current intensity (Figure 1B).

To record spontaneous excitatory synaptic currents (sEPSCs), the neurons were held at −70 mV in voltage clamp conditions. From the 200-s-long recordings the median amplitude, area (charge transfer), and average frequency of sEPSCs (Figures 1D-F) were calculated. Signals were analyzed with the NeuroExpress software written by A. Szűcs. The applied recording conditions and detection settings allowed the quantification of AMPA-mediated EPSCs at amplitude above + 7 pA.

### Field Potentials

**FIGURE 2:**
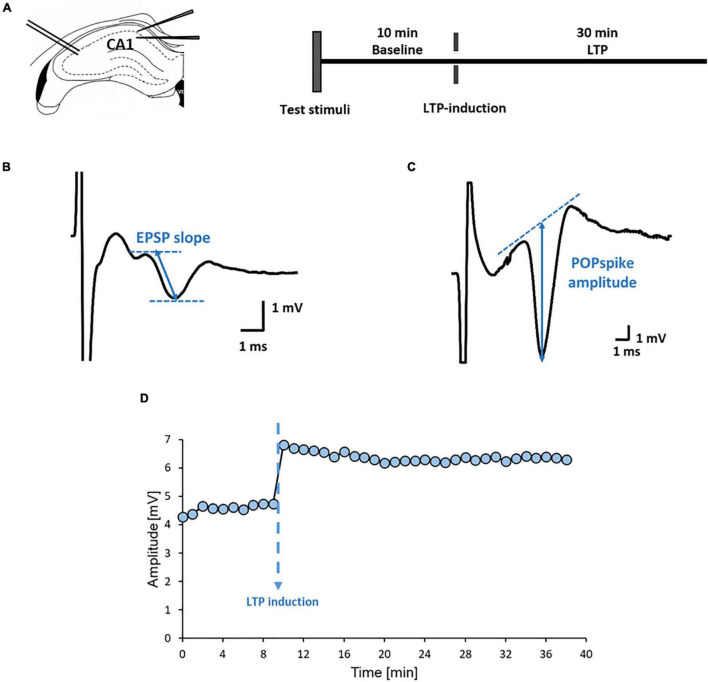
Experimental protocols used in field potential measurements. **(A)** Positioning of the electrodes for registering field potentials in the hippocampus: stimulation at the Schaffer-collaterals and recording in the stratum radiatum and the stratum pyramidale and the timeline of experiments for hippocampus slices. **(B)** Representative evoked EPSP recorded from stratum radiatum and **(C)** POPspike recorded from stratum pyramidale. **(D)** Representative plot of POPS amplitude over time during LTP experiments. After a 10-minute-long baseline, LTP was induced via theta burst stimulation, then, recording was continued for 30 min.

Field potential responses were evoked with a bipolar tungsten stimulation electrode placed at the Schaffer collaterals (square voltage pulses of 100 μs width, BioStim, Supertech Ltd., Pécs, Hungary) in the CA1 region. Two extracellular glass microelectrodes filled with 1 M NaCl (5-15 MΩ) were positioned into the *stratum radiatum* and the *stratum pyramidale* (Figure 2A) to record excitatory postsynaptic potentials (EPSPs) and population spikes (POP-spikes), respectively (Figures 2B,C).

Basic synaptic functions were tested by determining the voltage threshold of evoked field potentials (T). This was followed by testing long-term synaptic potentiation (LTP). Stimulus intensity was set up to evoke 70% of the maximal amplitude of POP-spikes. After recording a 10-min baseline, repetitive stimuli (theta burst stimulation, TBS) were applied with 10 bursts containing 4 pulses at 100 Hz repeated at 200 ms intervals (i.e., at 5 Hz) for inducing LTP. Then, recording was continued for 30 min (Figures 2A,D). Evoked field potentials were amplified (1,000×), filtered (0.16 Hz-2 kHz), recorded (sampling rate: 20 kHz) and analyzed with SPEL Advanced Intrasys computer program (Experimetria Ltd., Budapest, Hungary). Analyzed parameters were the initial slope of EPSPs (derived quantity from amplitude and rising time), the amplitude of POP-spikes (Figures 2B,C) and LTP% (the change in EPSP slope and POP-spike amplitude 30 min after LTP-induction compared to the baseline).

### Statistical Analysis

One-way ANOVA (with Levene’s test for homogeneity of variances and Tukey’s *post hoc* test, *p* < 0.05) was performed for statistical analysis to estimate the significance of differences between control and treated groups.

## Results

### Whole-Cell Patch Clamp Recordings

Physiological parameters extracted from the current step responses in current clamp configuration are shown in Table 1.

**TABLE 1:** Intrinsic membrane properties of CA1 pyramidal cells measured in current clamp experiments.

Current steps	Resting potential [mV]	Membrane resistance [MΩ]	Voltage sag index [mV/nA]	Spike threshold [mV]	Rheobase [pA]	Spike amplitude [mV]	Cumulative spike number [pcs]
**Male**							
**6-week-old**							
Control	−60.58 ± 0.72	92.41 ± 5.59	−9.82 ± 1.04	−39.35 ± 1.00	167.69 ± 11.47	90.06 ± 1.38	118.59 ± 20.27
Treated	−60.87 ± 1.15	95.42 ± 4.89	−10.93 ± 0.91	−38.84 ± 1.20	175.45 ± 16.35	85.33 ± 1.49	96.77 ± 22.25
**3-month-old**							
Control	−59.95 ± 0.67	116.71 ± 6.87	−9.88 ± 1.08	−41.49 ± 1.49	135.98 ± 10.62	83.55 ± 1.55	134.00 ± 17.19
Treated	−57.31 ± 0.62	123.40 ± 7.22	−12.09 ± 1.07	−41.36 ± 1.07	99.22 ± 12.19 *	87.10 ± 1.63	221.42 ± 32.06 *
**Female**							
**6-week-old**							
Control	−56.45 ± 0.64	99.68 ± 6.32	−5.44 ± 0.58	−35.45 ± 1.22	148.43 ± 16.43	82.14 ± 1.53	117.06 ± 25.38
Treated	−57.58 ± 0.83	101.33 ± 10.24	−10.00 ± 1.56 **	−38.73 ± 1.82	141.19 ± 21.66	82.35 ± 2.88	150.40 ± 40.07
**3-month-old**							
Control	−56.20 ± 0.82	113.13 ± 5.92	−8.30 ± 0.56	−36.59 ± 1.24	124.29 ± 12.90	82.14 ± 1.35	121.06 ± 15.48
Treated	−61.93 ± 1.34 ***	97.81 ± 5.06	−9.69 ± 0.69	−41.30 ± 1.14 *	151.59 ± 19.21	86.95 ± 1.23	118.25 ± 32.28

Data are shown as averages with standard errors. N = 19, 13, 22, and 19 in case of males and N = 16, 15, 18, and 16 in case of females in the 6-week-old control and treated and 3-month-old control and treated groups, respectively. Statistical analysis: one-way ANOVA; * means significant difference between the control and treated groups in the same age and sex; *p < 0.05; **p < 0.01; ***p < 0.001.

In general, CA1 pyramidal cells had a resting membrane potential around −60 mV and had a relatively low input resistance (around 100 MΩ). Injection of negative current steps revealed a small depolarizing voltage sag in their responses. The neurons typically displayed a regular firing pattern in response to positive current steps and the amplitude of the action potentials was higher than 80 mV.

**FIGURE 3:**
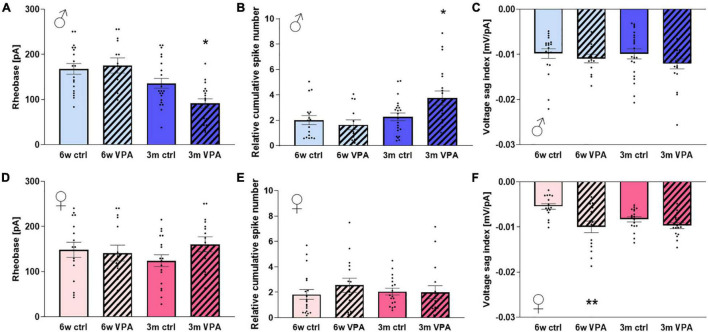
The alterations in the membrane properties of CA1 pyramidal neurons of 6-week-old and 3-month-old rats of both sexes exposed to VPA prenatally compared to the control. **(A)** The average rheobase and **(B)** cumulative spike number as a function of the input current and **(C)** voltage sag index in males. **(D)** The average rheobase and **(E)** cumulative spike number as a function of the input current and **(F)** voltage sag index in females. Data are expressed as mean and S.E.M. Statistical analysis: one-way ANOVA; * significant difference compared to control group; *: *p* < 0.05; **: *p* < 0.01.

In males we found no differences between the control and VPA-treated groups at the age of 6 weeks. However, in 3-month-old males the lower rheobase (Figure 3A) and the higher cumulative spike number seen in the VPA-treated group (Figure 3B) indicate the increased excitability of CA1 pyramidal cells. These parameters were not altered in females (Figures 3D,E).

In 6-week-old VPA-treated females, the voltage sag index was shifted to the negative direction compared to controls (Figure 3F). This observation indicates an overall upregulation of the voltage sag in a range of applied currents and suggests the increased function of hyperpolarization-activated non-specific cation channels mediating the h-current. However, this alteration was not detected in the 3-month-old treated females or in males (Figure 3C). In the latter group the membrane potential was more hyperpolarized compared to the control of the same age but interestingly, the spike threshold potential was also shifted in the negative direction resulting in unaltered spiking activity.

Parameters of spontaneous miniature excitatory postsynaptic currents (sEPSC) recorded in voltage clamp configuration are shown in Table 2.

**TABLE 2:** Parameters of EPSCs of CA1 pyramidal cells measured in voltage clamp configuration.

EPSC	Area [fC]	Amplitude [pA]	Frequency [Hz]
**Male**			
**6-week-old**			
control	88.49 ± 4.95	54.90 ± 8.23	0.52 ± 0.15
treated	59.21 ± 5.36	27.55 ± 1.44 ***	1.33 ± 0.39
**3-month-old**			
control	190.10 ± 13.84	25.94 ± 2.76	1.18 ± 0.24
treated	67.66 ± 2.44***	13.60 ± 0.94	4.19 ± 0.89 ***
**Female**			
**6-week-old**			
control	62.39 ± 5.61	28.17 ± 1.92	0.65 ± 0.21
treated	58.07 ± 2.91	30.52 ± 1.59	2.25 ± 0.69 *
**3-month-old**			
control	168.03 ± 10.79	19.61 ± 1.24	2.40 ± 0.60
treated	170.81 ± 6.36	20.39 ± 1.34	0.71 ± 0.18 *

Data are shown as averages with standard errors. N = 15, 14, 17, and 12 in case of males and N = 10, 11, 15, and 13 in case of females in the 6-week-old control and treated and 3-month-old control and treated groups, respectively. Statistical analysis: one-way ANOVA; * means significant difference between the control and treated groups in the same age and sex; *p < 0.05; ***p < 0.001.

**FIGURE 4:**
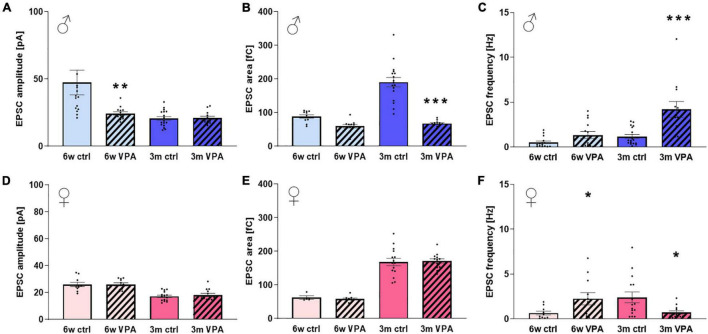
The parameters of spontaneous miniature excitatory postsynaptic currents (EPSCs) of 6-week-old and 3-month-old rats of both sexes exposed to VPA prenatally compared to the control. **(A)** The median of the amplitudes, **(B)** the area (charge transfer) and **(C)** the average frequency of EPSCs in males. **(D)** The median of the amplitudes, **(E)** and the area **(F)** the average frequency of EPSCs in females. Data are expressed as mean and S.E.M. Statistical analysis: one-way ANOVA; * significant difference compared to control group; *: *p* < 0.05; **: *p* < 0.01; ***: *p* < 0.001.

In treated 6-week-old males the mean amplitude of sEPSCs were smaller compared to the control (Figure 4A) suggesting the decrease in the postsynaptic response to the spontaneous presynaptic release of excitatory transmitters. This difference was not detected in the 3-month-old males. The area (transferred charge) of the EPSCs in treated 6-week-old males was not significantly different compared to the controls (Figure 4B), this result, together with the decreased amplitude suggests that EPSCs had a slower kinetics in this treated group. In case of 3-month-old treated males, a decreased EPSC area was observed compared to controls, while the amplitude was unchanged, suggesting more rapid kinetics in the treated group. In control males, an age-dependent slowing of EPSC kinetics can be observed as indicated by the decrease of amplitude and parallel increase of the EPSC area, while in VPA-treated rats, there is no difference between the two age groups.

In the case of female rats, VPA treatment did not modify the amplitude (Figure 4D) or the area (Figure 4E) of EPSCs. In contrast, a decrease in amplitude and increase in area could be observed with age, suggesting a slowing kinetics during ontogenesis, similarly to male animals.

As for the frequency of sEPSCs, in 6-week old males, VPA treatment did not alter this parameter, while it increased markedly in the 3-month-old male treated group compared to the control (Figure 4C). This indicates the increase of probability of presynaptic spontaneous release of excitatory transmitters. In females the frequency of sEPSCs of the treated group was increased in VPA-treated 6-week-old rats compared to controls (Figure 4F) which, however, was not only compensated but even decreased by the age of 3 months where lower frequency could be seen in treated rats. No clear age dependence could be demonstrated for EPSC frequency, but a tendency of increase can be discerned in both sexes from 6 weeks to 3 months of age.

### Field Potentials

Parameters of evoked field potentials are shown in Table 3.

**TABLE 3:** Parameters of evoked field potentials measured in the CA1 region of HC.

Field potentials	EPSP threshold [V]	EPSP slope [mV/s]	EPSP LTP [%]	POPspike threshold [V]	POPspike amplitude [mV]	POPspike LTP [%]
**Male**						
**6-week-old**						
Control	2.75 ± 0.19	1.78 ± 0.27	140.20 ± 13.93	3.25 ± 0.30	5.75 ± 0.31	128.23 ± 8.67
Treated	2.14 ± 0.18 *	2.00 ± 0.31	138.15 ± 18.59	2.50 ± 0.11 **	7.25 ± 0.41 *	138.79 ± 15.35
**3-month-old**						
Control	2.13 ± 0.08	2.35 ± 0.27	136.24 ± 4.10	2.56 ± 0.11	6.97 ± 0.50	145.17 ± 7.69
Treated	1.94 ± 0.11	1.87 ± 0.27	142.84 ± 23.97	2.13 ± 0.13	6.97 ± 0.72	129.40 ± 15.89
**Female**						
**6-week-old**						
Control	2.25 ± 0.13	2.10 ± 0.53	135.50 ± 8.88	2.63 ± 0.13	5.60 ± 0.25	145.54 ± 8.47
Treated	2.42 ± 0.20	1.87 ± 0.58	126.61 ± 16.83	3.00 ± 0.13	5.54 ± 0.49	123.22 ± 8.57
**3-month-old**						
Control	2.69 ± 0.28	2.10 ± 0.23	144.24 ± 14.14	3.00 ± 0.33	7.39 ± 0.69	124.41 ± 19.40
Treated	2.00 ± 0.00 *	2.12 ± 0.35	134.44 ± 9.89	2.31 ± 0.13	7.12 ± 0.38	112.57 ± 14.53

Data are shown as averages with standard errors. N = 6, 6, 8, and 8 in case of males and N = 8, 6, 8, and 8 in case of females in the 6-week-old control and treated and 3-month-old control and treated groups, respectively. Statistical analysis: one-way ANOVA; * means significant difference between the control and treated groups in the same age and sex; *p < 0.05; **p < 0.01.

**FIGURE 5:**
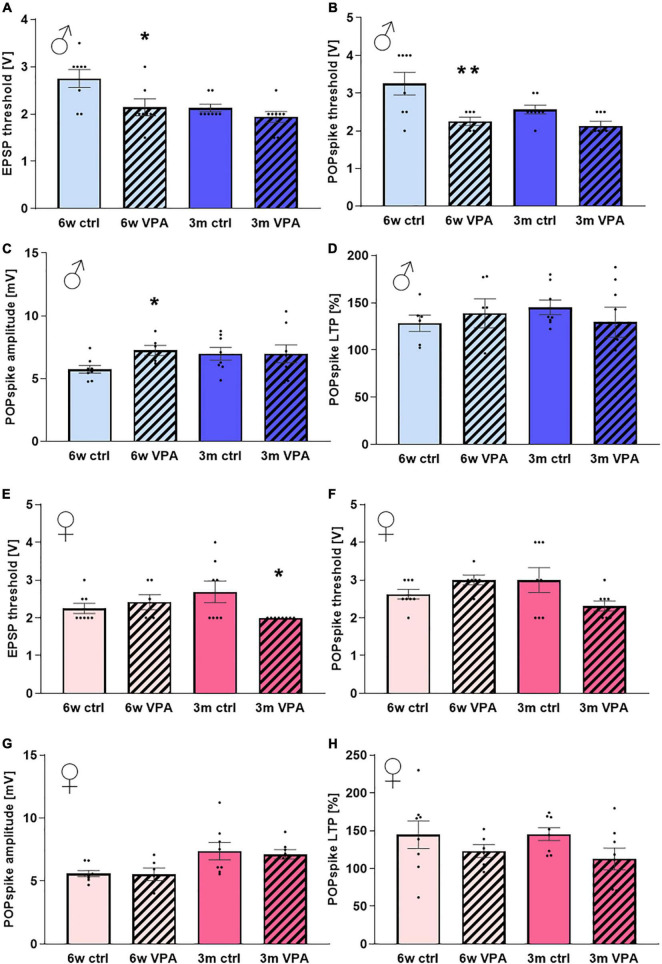
The effects of prenatal VPA-treatment on hippocampal neuronal networks of 6-week-old and 3-month-old rats of both sexes. **(A)** The average threshold of evoked field EPSPs and **(B)** POPspikes, **(C)** the average amplitude of POPspikes and **(D)** the average change in POPspike amplitude 30 min after LTP-induction compared to the baseline in males. **(E)** The average threshold of evoked EPSPs and **(F)** POPspikes, **(G)** the average amplitude of POPspikes and **(H)** the average change in POPspike amplitude 30 min after LTP-induction compared to the baseline in females. Data are expressed as mean and S.E.M. Statistical analysis: one-way ANOVA; * significant difference compared to control group; *p < 0.1; **p < 0.005.

In 6-week-old treated males the basic hippocampal network excitability was enhanced drastically as the threshold of both EPSPs and POPspikes decreased (Figures 5A,B) while the amplitude of POPspikes was elevated (Figure 5C). These differences, however, were not shown in 3-month-old males.

In females the only alteration was found in case of the 3-month-old treated group, where the EPSP threshold decreased (Figures 5E-G).

**FIGURE 6:**
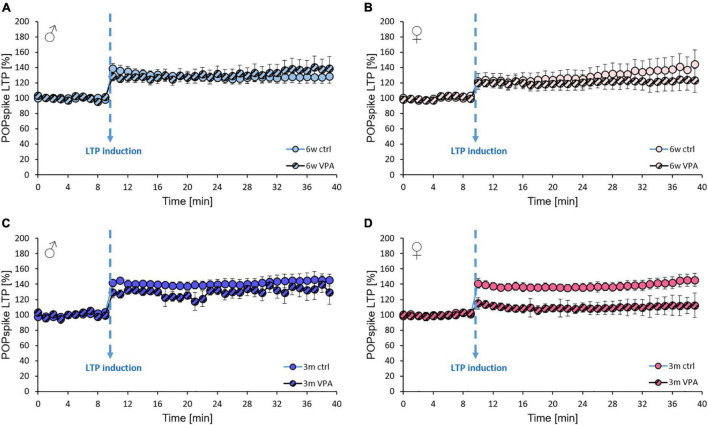
The effects of prenatal VPA-treatment on the LTP of hippocampal neuronal networks in 6-week-old and 3-month-old rats of both sexes. **(A)** The average change in POPspike amplitude expressed as % of the 10-min baseline (time points were taken as an average of 6 data points) in control and VPA-treated 6-week-old males and **(B)** females and **(C)** in 3-month-old males and **(D)** females. Arrow indicates the time point of theta burst stimulation (TBS). LTP induction was followed for 30 min after TBS. Data are expressed as mean and S.E.M. Statistical analysis: repeated-measures ANOVA.

Although, the development of LTP was also investigated in the current study, there were no changes detected in the degree of long-term enhancement of evoked potentials in any of the treated groups after 30 min (Figures 5D,H). The process of the POPspike amplitude enhancement after TBS was similar in case of control and VPA-treated 6-week-old (Figure 6A) and 3-month-old (Figure 6C) males. In case of females, there was a tendency for a lower enhancement in slices originating from VPA-treated rats compared to age-matched controls, in both the 6-week-old (Figure 6B) and 3-month-old (Figure 6D) age group, but these differences were not statistically significant.

## Discussion

To summarize, a variety of alterations in hippocampal network and neuronal functions were observed depending on the age and sex of the animals prenatally exposed to VPA.

In 6-week-old males, the amplitude of the sEPSCs recorded on CA1 pyramidal cells decreased compared to the control, while their area was unchanged, suggesting a slower kinetics of EPSCs, which indicates a decrease in number or altered subunit composition of the glutamate receptors. These changes may be a homeostatic “synaptic scaling” as a response to an enhanced network activity to maintain synaptic strength in a compensatory manner (Galanis and Vlachos, 2020). This idea is also supported by our other observation that the threshold of the evoked field potentials decreased significantly with an increased POPspike amplitude, meaning the elevated number of firing neurons surrounding the electrode. However, the enhanced network activity cannot be explained only with the increased intrinsic activity of pyramidal cells, since we did not observe differences between the membrane properties of the cells of the control and treated groups. In contrast with our data, in an experiment studying the membrane properties of 45-day-old male VPA-treated rats, the rheobase decreased and the frequency of evoked firing increased, assuming a highly increased excitability of pyramidal cells in the CA1 region (Hajisoltani et al., 2019). It should be noted that in these experiments the stimulation protocols were different compared to ours. Therefore, it would be beneficial to investigate the interneurons since GABAergic system dysregulation, including decreased number of GABAergic interneurons and impaired GABAergic neurotransmission, has been proposed as a source of E/I imbalance in the HC, as well. To support this idea, the expression of glutamic acid decarboxylase (GAD67), an enzyme responsible for the synthesis of GABA from glutamate (Puig-Lagunes et al., 2021), was measured in different brain areas of VPA-treated young and adolescent male rats. As suspected, significant decrease in the GAD67 levels was found in the HC in all age groups investigated from postnatal day 7 and 72 (Hou et al., 2018). Moreover, the frequency of spontaneous miniature inhibitory postsynaptic currents decreased in adolescent rats suggesting the decreased probability of GABA release of the neighboring cells (Banerjee et al., 2013). In another study, the effect of prenatal VPA treatment was systematically examined on hippocampal circuit development in male rat pups between postnatal day 13 and 18 with field potential recording. At this young age, POPS amplitudes were higher in treated pups on certain days, showing that VPA treatment accelerates neuronal excitability increase during ontogenesis, presumably by influencing GABA levels (Fueta et al., 2018).

The alterations observed by us in 6-week-old male rats were not shown in the 3-month-old males, however, the frequency of sEPSCs increased markedly in the treated group with no changes in evoked field potential parameters, indicating unaltered network excitability. Furthermore, increased excitability of the neurons is assumed due to the decreased rheobase leading to an elevated cumulative spike number. In the CA1 and CA3 regions of HC the loss of pyramidal cells was reported (Hou et al., 2018) which might force the increased activity of the fewer cells to maintain the functions of the network. According to a study investigating the prefrontal cortex and the cerebellum of non-human primates, VPA inhibits neurogenesis, disrupts network formation and promotes astrocyte production during embryo development (Zhao et al., 2019). Furthermore, in the HC increased glutamate (Glu) concentrations were reported in rodents (Zieminska et al., 2018) which is consistent with the detection of significantly higher levels of Glu in HC among several brain areas of ASD patients (Puig-Lagunes et al., 2021).

In 6-week-old treated females, the voltage sag index was shifted in the negative direction compared to the control suggesting that the cells were more resistant against hyperpolarizing current stimuli (shunting), however, the probability of after depolarization or even post-inhibitory after discharge with the cessation of the negative current steps increases (Rátkai et al., 2021). In addition, the frequency of sEPSCs increased, while the amplitude of sEPSCs remained unaltered and there were no other changes in the membrane properties assuming homeostatic adaptation to the increased probability of transmitter-release from the neighboring cells. Moreover, there were no differences in the network excitability between the treated and control animals. It is plausible that this is the result of pyramidal cell loss mentioned above (Hou et al., 2018). It is interesting though, that in case of males this increase in sEPSC frequency occurred in the 3-month-olds while there were no changes indicating the decreased number of pyramidal cells in the younger animals like in case of female ones. In a study measuring the plasma concentration of 14-day-old rats, it was reported that Glu levels were higher in the HC of female rats exposed to VPA compared to controls but there were no such changes in case of males (Puig-Lagunes et al., 2021). However, other experiments suggest that the concentration of Glu is increased in male HC after VPA-treatment in 31-day-old rats as well (Zieminska et al., 2018). Based on these findings, it is possible that these changes occur in an earlier phase of the development in females compared to males. These differences are supported by several human studies suggesting sex differences in the developmental trajectories of the human hippocampus, including faster rates of hippocampal growth for female participants (Reinhardt et al., 2020).

In the 3-month-old females, the membrane potential in the treated group was shifted to the negative direction, although it had no effect on the firing due to the shift of the spike threshold in the same direction. Interestingly, the frequency of the EPSCs was not only compensated by this age but it decreased compared to the control group. Nevertheless, on the network level, increased excitability was demonstrated, as seen in the significant decrease of the EPSP threshold in the treated group. Since there were no changes in the membrane properties of the pyramidal cells indicating increased excitability, it may be helpful for the discussion to investigate the functions of the GABAergic system.

Long-term synaptic plasticity was also studied by means of LTP induction in the CA1, and no differences in LTP efficacy were observed in either males or females in either age group. As in our study, increased network excitability was demonstrated in 6-week-old male VPA-treated animals, indicated by decreased stimulation thresholds and enhanced POPS amplitudes, enhanced LTP might have been expected in this group. At the Schaffer collateral-CA1 synapse, long-term synaptic enhancement is basically dependent on NMDA receptor functions. Plasticity alterations, namely hyper-plasticity have been reported in the VPA autism model in two-week-old rats in another brain area, the prefrontal cortex, along with NMDA receptor overexpression (Rinaldi, 2008). However, there are no data about hippocampal LTP in the VPA autism model. In a different treatment paradigm, applying chronic prenatal VPA treatment, significant decrease in hippocampal LTP was demonstrated in 3-4 week-old rats (Zhang et al., 2003). However, the comparison with our results is doubtful, because the stimulation thresholds and field potential amplitudes were not notified in that study. In a different model of autism, the possible contribution of certain cell membrane receptors, like receptor tyrosine kinase is emphasized to explain altered plasticity: tyrosine kinase receptors activate excitatory processes enabling normal long term synaptic efficacy changes. These receptors are also affected by the valproate treatment (Ma et al., 2019).

Hippocampal changes on the cellular or on the network level may cause different behavioral alterations. It is well known that the dorsal HC is important for spatial learning and memory and the ventral HC is essential for emotional behavior (Li et al., 2019). Several MRI studies investigated the changes in the HC volume of ASD patients, however, these findings are not consistent. It is a popular view that individuals with ASD have generally enlarged hippocampi, but this is true for only a subset of the patients (Reinhardt et al., 2020). There is a possibility that the increased size of the HC indicates a use-dependent expansion of hippocampal connections which might suggest a hyper-function of this structure, thus one could expect enhanced spatial or episodic memory task performance in autism. This idea is supported by the findings that there are people with autism having extraordinary capability of learning a route on a map (Edalatmanesh et al., 2013). Regarding the animal models of autism different data can be found on the altered spatial learning and memory tested in Morris Water Maze. There are experiments supporting the idea that VPA-treated rats learn to find the platform more effectively than control animals (Edalatmanesh et al., 2013), however, in other experiments treated rats seemed to have delayed memory acquisition skills and to be resistant to sudden changes in memory tasks (Hou et al., 2018). Like autistic patients, VPA-treated animals can also differ according to the affected brain regions and in the severity of the symptoms, which may explain the contradictory findings in the Water Maze experiments. Nevertheless, the above-described failure of the animals to quickly adapt to the formation of new memories may be analogous to the inflexibility in routine that is characteristic of autistic patients (Hou et al., 2018). Furthermore, it is suggested that anxiety-like behavior is related to the decrease in dendritic spine density in the CA1 region of HC, which can be observed in several psychiatric disorders and also in VPA-treated mice (Yamaguchi et al., 2017). According to other authors the excitatory-inhibitory imbalance observed in the HC also plays a pivotal role in anxiety-like behavior (Hou et al., 2018; Khodaverdi et al., 2021). Children with autism can experience anxiety more often and more intensely than healthy children; it is not surprising then, that VPA-treated rats also displayed enhanced anxiety-like behaviors in the open field and elevated plus maze tests (Hou et al., 2018; Khodaverdi et al., 2021) presumably as a result of the deterioration of the connection between the ventral HC and the medial prefrontal cortex (Hou et al., 2018).

Altogether, the early changes in the nervous system may be compensated or new alterations may develop, even in the young adult rats, and developmental trajectories may be different in males vs. females. These age differences have been observed before, for example in case of the nociception threshold, which was shown to decrease in juvenile rats, but increase in adult ones (Zieminska et al., 2018). Age-related morphological abnormalities in the cerebellum of rats and cortical thinning in the temporal lobe in human studies were observed, as well (Spisák et al., 2019). In our study, in the hippocampus of treated animals there were robust changes at the age of 6 weeks compared to the control which were not shown in the 3-month-olds, yet other impairments arised. Moreover, different alterations were observed depending on the sex of the animals, this has also been demonstrated previously in literature data. Sex-specific impairments were found in social behavior of VPA-treated animals as exposed males generally ignored stranger conspecific while exposed females spent more time sniffing the stranger rat (Cho et al., 2017; Scheggi et al., 2020). Furthermore, only male animals displayed a lower sensitivity to pain and a decreased level of social interactions, however, both sexes exhibited increased repetitive behavior. In addition, cell loss in the cortex was also sex-specific, cell loss in the somatosensory cortex was only detected in male rats (Cho et al., 2017).

To the best of our knowledge, this is the first study examining hippocampal changes in the rodent VPA autism model on both sexes in two age groups in young adult age. Our results indicate various changes in network functions and pyramidal neuron properties. Further studies investigating GABA-ergic functions and eventual structural alterations would elucidate the exact mechanism of VPA effect in the hippocampal network.

## Data Availability Statement

The raw data supporting the conclusions of this article will be made available by the authors, without undue reservation.

## Ethics Statement

The animal study was reviewed and approved by Budapest Animal Health Care Authority.

## Author Contributions

VB, PV, IV, and AS designed the study. VB, TM, and VK performed the experiments and analyzed the data. All authors contributed to the edition and revision of the manuscript.

## Conflict of Interest

The authors declare that the research was conducted in the absence of any commercial or financial relationships that could be construed as a potential conflict of interest.

## Publisher’s Note

All claims expressed in this article are solely those of the authors and do not necessarily represent those of their affiliated organizations, or those of the publisher, the editors and the reviewers. Any product that may be evaluated in this article, or claim that may be made by its manufacturer, is not guaranteed or endorsed by the publisher.
